# Supplementary material for: Causal interplay between lactose intolerance and gut microbiota: a combined bidirectional Mendelian randomization and in vivo validation study
Source: Front Nutr. 2026 Jun 1;13:1803337. doi: 10.3389/fnut.2026.1803337 (PMC13265576; doi:10.3389/fnut.2026.1803337)
Supplement: Supplementary file 1 [file Data_Sheet_1.zip › supplementary materials/Forward/leave one out cross validation/ebi-a-GCST90027618.finngen_R12_E4_LACTONAS.pdf]

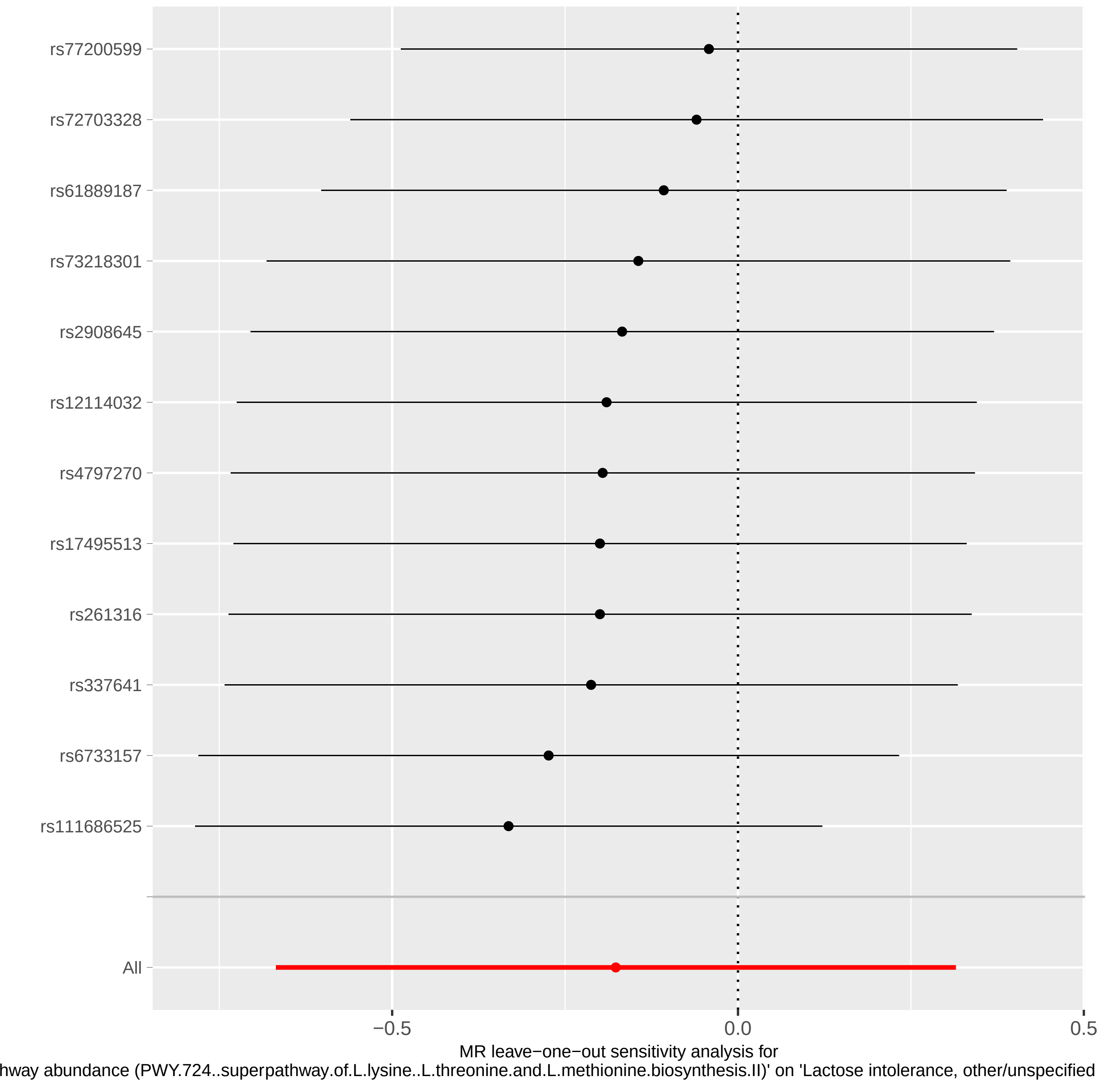

rs77200599
rs72703328
rs61889187
rs73218301
rs2908645
rs12114032
rs4797270
rs17495513
rs261316
rs337641
rs6733157
rs111686525
All
−0.5
0.0
0.5
MR leave−one−out sensitivity analysis for
hway abundance (PWY.724..superpathway.of.L.lysine..L.threonine.and.L.methionine.biosynthesis.II)' on 'Lactose intolerance, other/unspecified
